# Mechanism and Multilayer Perceptron prediction model of the removal of *α*-terpineol, terpinen-4-ol and carvone from pasteurized citrus juices by *β*-cyclodextrin encapsulation

**DOI:** 10.3389/fnut.2025.1557934

**Published:** 2025-05-21

**Authors:** Ruijie Mai, Shuo Xue, Jingnan Ren, Gang Fan, Jinchu Yang, Linhua Huang, Guijie Li, Yujiao Cheng, Qiuling Wang, Yongfeng Yang, Zhenzhen Huang, Yingjie Feng, Wenzhao Liu, Aiqun Yu, Jian Feng

**Affiliations:** ^1^Key Laboratory of Environment Correlative Dietology, Ministry of Education, College of Food Science and Technology, Huazhong Agricultural University, Wuhan, China; ^2^Technology Center, China Tobacco Henan Industrial Co., Ltd., Zhengzhou, China; ^3^Citrus Research Institute, Southwest University, Chongqing, China; ^4^State Key Laboratory of Food Nutrition and Safety, College of Biotechnology, Tianjin University of Science and Technology, Tianjin, China; ^5^Henan Jinrui Flavoring and Essence Co., Ltd., Zhengzhou, China

**Keywords:** citrus, off-flavor compounds, *β*-cyclodextrin, GCMS, SEM, TGA, molecular docking, deep learning

## Abstract

This study revealed the mechanism of improvement in sensory evaluation of citrus juices after pasteurization by the natural cyclodextrin (*β*-CD), and developed a prediction model for encapsulation based on conventional physicochemical indicators. The results of gas chromatography–mass spectrometry indicated that the off-flavor in citrus juice after pasteurization were mainly caused by *α*-terpineol, terpinen-4-ol and carvone, and the addition of *β*-CD could effectively reduce the content of these compounds. The inclusion complexes of *β*-CD and off-flavor compounds were characterized by formation constants, scanning electron microscope, X-ray diffraction, Fourier transform infrared spectroscopy, and thermogravimetric analyses, followed by molecular docking to show the possible conformations of *β*-CD and off-flavor compounds to form 1:1 inclusion complex by hydrophobic interaction, van der Waals forces and hydrogen bonding. Multiple prediction models were constructed and evaluated by deep learning using basic physicochemical indicators such as sucrose content, citric acid content, pH, temperature and storage conditions as input variables, and peak area of off-flavor compounds as output layer. The results showed that the Multilayer Perceptron Model had great potential in predicting the cyclodextrin embedding effect.

## 1 Introduction

Citrus is a member of the Rutaceae family whose fruits are flavorful and rich in a wide range of nutrients, including proteins, sugars, organic acids, flavonoids, many vitamins and mineral elements (1). Citrus juice is one of the main processed varieties of citrus. Sterilization is an essential operation in the production of citrus juice. However, heat treatment can have some adverse effects on the aroma, color, taste and nutrient content of citrus juice. Among them, aroma is one of the most important factors in measuring the quality and consumer acceptance of citrus juices. On the one hand, some of the aroma active compounds (mainly terpenes, aldehydes, and esters) in the juice evaporate during the heating process, and on the other hand, some off-flavor compounds (mainly terpenes degradants, sulfur-containing compounds, phenols, acids, and others) are newly generated in the juice during the heating process (2, 3). It has been shown that *α*-terpineol, terpinen-4-ol and carvone from the degradation of D-limonene and linalool were responsible for the off-flavor of citrus juice after heat treatment (4–7).

In order to reduce the production of off-flavor compounds, alternative processing techniques including microwave processing, ultrasound processing, hydrostatic pressure processing, electrical treatments and chemical treatment are used to inactivate enzymes and micro-organisms instead of conventional sterilization methods (8). However, these technologies are still difficult to industrialize on a large scale. Therefore cyclodextrins (CDs) become an attractive, cost-effective and safe strategy (9, 10). Cyclodextrins are able to form inclusion complexes with hydrophobic molecules or hydrophobic portions of compounds in aqueous environments due to their hydrophilic outer and hydrophobic inner surfaces, making cyclodextrins potentially advantageous for controlling flavor release or adsorption of off-flavor compounds (9, 11). Due to the small molecular cavity, *α*-CD can generally only encapsulate some smaller guest molecules. And although *γ*-CD has a large molecular cavity and can encapsulate larger guest molecules, its production cost is too high. Therefore *β*-CD, which has a suitable molecular cavity size and relatively low production cost, has been widely used (12).

Deep learning is an advanced machine learning technology that uses deep neural networks to simulate the learning process of the human brain, and automatically extracts the higher-order features of the data through multilayer nonlinear transformations to achieve modeling and prediction of complex problems (13). Food, as an intrinsically connected and complex whole, contains a wide range of components with different properties, ranging from basic metrics that are easy to measure (e.g., pH) to metrics that require complex instrumentation to accurately determine (e.g., flavor compounds). Given the costly and time-consuming nature of complex metrics, machine learning is a powerful tool for predicting complex metrics from basic metrics. In the field of food analysis, deep learning can be applied to food image recognition, composition analysis, and quality assessment (14). However, there are no studies using deep learning to predict the effect of cyclodextrin encapsulation under different conditions.

Improvement of food flavor properties by cyclodextrins has been reported, but there was little literature on the mechanisms by which cyclodextrins improve the flavor properties of foods, especially the mechanisms of interaction with these off-flavor compounds (*α*-terpineol, terpinen-4-ol and carvone), and the evaluation of their applications. This study aims to evaluate the effect of cyclodextrin treatment on the sensory evaluation, flavor compounds, physicochemical indexes and nutrients of citrus juice, and further demonstrated the ability of cyclodextrins to form encapsulation with three off-flavor compounds (*α*-terpineol, terpinen-4-ol and carvone) through characterization, and finally evaluated the encapsulation effect of cyclodextrins on the off-flavor compounds under different conditions and constructed a physicochemical index-based encapsulation prediction model.

## 2 Materials and methods

### 2.1 Materials

The mandarin oranges (*Citrus reticulata*) were supplied by the Yunnan Comprehensive Experimental Station for Very Early Citrus of the National Citrus Industry Technology System (Yunnan, China). The cyclodextrin (*β*-CD) was purchased from Shanghai Yuanye Bio-Technology Co., Ltd. (China) and the off-flavor compounds (*α*-terpineol, terpinen-4-ol and carvone) were purchased form Sigma-Aldrich (USA).

### 2.2 Effect of *β*-CD on the citrus juice

#### 2.2.1 Sensory evaluation

Citrus juice was obtained from fresh mandarin oranges by peeling and squeezing, filtered through 80-mesh sterile double-layered gauze, and placed in a refrigerator at 4°C for use. A portion of the citrus juice was pasteurized (85°C for 15 min). The *β*-CD was added to the pasteurized citrus juice and the concentrations were adjusted to 2, 4, 6, 8 and 10 mmol/L. Sensory testing was undertaken referring to the method reported by Mai et al. (15). Twelve trained panelists (6 males and 6 females; aged between 22 and 28) were chosen for this sensory test based on Quantitative Descriptive Analysis (QDA). Aroma, taste and color were generated in a seminar. Randomized samples were assessed at the same time. In a test room (25°C), the panelists were asked to express their sensory evaluation scores on a scale (1–9 for low to high).

#### 2.2.2 Extraction and identification of volatile compounds

The volatile compounds in citrus juice were separated by solid-phase micro-extraction (SPME) and following identified by gas chromatography–mass spectrometry (GC–MS) according to the method described by Ma et al. (16). A total 100 μL of internal standard (cyclohexanone; ≥ 99%; China National Medicines Corporation Ltd., China) and 3.6 g of sodium chloride (China National Medicines Corporation Ltd., China) were added into each citrus juice (20 mL). Then the citrus juice was maintained at 40°C for 15 min and a fiber sampler was inserted to adsorb the volatile compounds for 40 min. The fiber sampler was resolved at 250°C for 5 min. The GC–MS system (6,890 N -5977B; Agilent, USA) coupled with a HP-5MS column (30 m × 0.25 mm × 0.25 μm; Agilent) were used for identification of volatile compounds. The column was first held at 40°C for 3 min. An increasing rate of 3°C/min was applied to the temperature until reaching 160°C and held for 3 min; then increased to 160°C at a rate of 3°C/min. It finally increased to 220°C at a rate of 8°C/min and held for 2 min. The carrier gas (helium) flow rate was 1.2 mL/min. Mass spectra conditions: ion source temperature for 230°Cand EI voltage for 70 eV; scan range of 50–550 m/z. NIST05.L was used for the identification of volatile compounds. The concentration of the volatile compounds was calculated based on the equation below:


$$\text{Concentration of volatile compounds}\;(\mathrm{\mu g}/\mathrm{mL})=\frac{P\times Z}{M}$$

Where P was the ratio of the peak of volatile compounds to the peak area of the cyclohexanone, and Z and M were the quality of the cyclohexanone and the sample, respectively. OAV was the ratio of the concentration of volatile compounds to its threshold (μg/mL).

#### 2.2.3 Determination of the physicochemical indexes and nutrients

The pH in citrus juice was determined using pH meter method in Chinese National Standard GB/T 10468–1989. The titratable acid content and soluble solids content in citrus juice was determined using the indicator method and Abbe refractometer method in Chinese National Standard GB/T8210-2011, respectively. The vitamin C content in citrus juice was determined using the 2,6-dichloroindophenol titration method in Chinese National Standard GB6195-1986. The flavonoid content in citrus juice was determined using the colourimetric method in Chinese National Standard NY/T 2010–2011.

### 2.3 Interactions between *β*-CD and off-flavor compounds

The three guest molecules were added into the *β*-CD solution separately and their molar ratio was adjusted to 1:1. The obtained mixtures were shaken at 300 r/min for 24 h at 25°C, followed by standing at 4°C for 24 h. Finally, *β*-CD and the three guest molecules and inclusion complexes were obtained by freeze-drying.

The physical mixtures of *β*-CD and three guest molecules were obtained by adding *β*-CD and three guest molecules in a molar ratio of 1:1 (10 mmol), respectively, to an agate mortar and grinding them well.

#### 2.3.1 Determination of retention rate and formation constant

A total 1 μL of guest molecules (*α*-terpineol, terpinen-4-ol and carvone; Sigma-Aldrich, USA) were added to 100 mL of prepared *β*-CD solutions at concentrations of 0, 1, 4, 7 and 10 nmol/L, respectively. After sealing, the samples were shaken at 25°C for 30 min at 200 r/min. A total 10 mL of the sample was used for SPME-GC–MS. A total 10 mL of sample was analyzed using the SPME-GC–MS conditions described above. The retention rate was calculated from Ciobanu et al. (17).


$$R\;(\%)=\frac{1-A_{\mathrm{CD}}}{A_{0}}$$

Where A_CD_ and A_0_ were the peak area of the guest molecules in guest molecules measured with and without the addition of 10 mol cyclodextrin, respectively, and R was the retention rate.

The formation constant was calculated with reference to Ciobanu et al. (17) and Blach et al. (18). The formation of inclusion complexes between *β*-CD and guest molecules in aqueous solution was a dynamic equilibrium process. Assuming a binding ratio of 1:1, the inclusion equilibrium constant (K) was given by:


$$K=\frac{\left[A-\mathrm{CD}\right]}{\left[A]\times [\mathrm{CD}\right]}$$

Where [A], [CD], and [A-CD] were the equilibrium concentrations of the guest molecule, *β*-CD, and its envelope, respectively. Assuming a starting *β*-CD concentration of [CD]_0_ and a starting concentration of [A]_0_ for guest molecule, Equation 1 could be written as:


(1) $$K=\frac{{\left[A\right]}_{0}-[A]}{\left[A]\times [\mathrm{CD}\right]}$$

Since the amount of *β*-CD used was much larger than the amount of guest molecule so [CD]_0_ ≈ [CD], equation (2) could be written as:


$$K=\frac{{\left[A\right]}_{0}-[A]}{[A]\times {\left[\mathrm{CD}\right]}_{0}}$$

So from Equation 1 it could be introduced:


$$\frac{{\left[A\right]}_{0}}{\left[A\right]}=1+K\times [\mathrm{CD}]$$

where K was K_f_. The formation constant K_f_ of the guest molecule to *β*-CD could be found using [A]_0_/[A] as the vertical coordinate and the starting concentration of *β*-CD as the horizontal coordinate.

#### 2.3.2 Determination of embedding rate

The three guest molecules were added to anhydrous ethanol and their concentration were adjusted by 0.1, 0.2, 0.3, 0.4, 0.5, and 0.6 μL/ mL. The calibration curves were calculated by analyzing the peak area using gas chromatography (GC) according to the method described by Ma et al. (16). The gas chromatography system (6,890 N; Agilent) coupled with a HP-5 column (30 m × 0.32 mmol × 0.5 μm; Agilent) were used for the detection of the guest molecules. The flow rate of nitrogen was 1.0 mL/min. The diversion ratio was 50:1. The column was first held at 40°C for 1 min then increased to 220°C at a rate of 6°C/min and maintained for 1 min. Finally, the temperature was increased to 250°C at a 30°C/min rate. A total 20 mg of the inclusion complex was added to 5 mL of anhydrous ethanol, sealed and sonicated at 60°C for 30 min, which allowed the transfer of the guest molecules from the cavities of the cyclodextrins into anhydrous ethanol. The samples were processed by centrifugation at 4200 r/ min for 5 min to obtain the supernatant, which was filtered and then analyzed by gas chromatography peak area and the content was calculated using the calibration curves. Embedding rate was calculated with reference to Tian et al. (19).


$$E(\%)=\frac{M_{1}}{M_{2}}$$

Where M_1_ and M_2_ were the amount of guest molecules in the inclusion complex (mol) and the amount of guest molecules initially invested (mol), respectively.

#### 2.3.3 Scanning electron microscope (SEM)

SEM (MERLIN Compact, Carl Zeiss AG, Germany) was used to obtain clearer morphology and structure of *β*-CD and three inclusion complexes. After gold sputtering under high vacuum, the samples were observed at 3.0 Kv with magnifications of 600x and 3,000x, respectively.

#### 2.3.4 X-ray diffractometer (XRD)

XRD (D8 ADVANCE, Bruker Corporation, Germany) was used to characterize the crystalline state of *β*-CD, three physical mixtures, and three inclusion complexes. Cu Kα rays were used with tube voltage and current of 40 kV and 200 mA, respectively. Scanning range of 2θ was 3° to 40°, scanning rate was 8°/min, and step size was 0.02°.

#### 2.3.5 Fourier transform infrared spectrometer (FT-IR)

FT-IR (Nexlus, Mettler-Toledo International Inc., Switzerland) was used to characterize the structural changes in *β*-CD, three physical mixtures, and three inclusion complexes. Potassium bromide tablet method was for *β*-CD, three physical mixtures, and three inclusion complexes, and potassium bromide film method was for *α*-terpineol, terpinen-4-ol, and carvone. The wavenumber range was 4,000–400 cm^−1^.

#### 2.3.6 Thermogravimetric analysis (TGA)

Thermogravimetric analyzer (Mettler-Toledo International Inc., Switzerland) was sued for *β*-CD, three physical mixtures, and three inclusion complexes. The temperature range was 40–500°C and the temperature increase rate was 10°C/min.

#### 2.3.7 Molecular docking

The molecular structures downloaded from ChemSpider^1^ were geometrically optimized by ChemBio3D 14.0 (PerkinElmer Inc., USA), and molecular simulations were docked with AutoDock 4.2.6 (The Scripps Research Institute, Olson Lab, USA) based on previously reported literature (20, 21).

### 2.4 Encapsulation effect of *β*-CD on the off-flavor compounds under different conditions

The mother liquor of off-flavor compounds was prepared by dissolving 100 μL of each of three off-flavor compounds (*α*-terpineol, terpinen-4-ol and carvone; Sigma-Aldrich, USA) in 10 mL of ethanol. The *β*-CD were dissolved in distilled water and prepared into cyclodextrin solutions at concentrations of 2, 4, 6, 8, and 10 mmol/L, respectively.

#### 2.4.1 Sucrose and citric acid

Sucrose or citric acid (Sinopharm, China) was added to the cyclodextrin solutions (10 mL; 10 mmol/L) and their concentrations were adjusted to 2, 4, 6, 8, 10 and 0.2%, 0.4, 0.6, 0.8, 1.0%, respectively. Then 10 μL of the mother liquor (*α*-terpineol, terpinen-4-ol and carvone; 10 μg/mL) was added. The off-flavor compounds were extracted by SPME-GC.

#### 2.4.2 pH

A total 100 mL of buffer solutions with pH values of 3, 5, 7, 9 and 11 were prepared according to Chinese National Standard GB/T 601–2002 and two cyclodextrins were added and adjusted to a concentration of 10 mmol/L. Then 10 μL of the mother liquor was add and the off-flavor compounds were extracted and detected by SPME.

#### 2.4.3 Temperature

The cyclodextrin solutions (10 mmol/L) was heated to 20, 40, 60 and 80°C, and the mother liquor (10 μL) was added. The off-flavor compounds were extracted and detected by SPME-GC.

#### 2.4.4 Storability

The mother liquor (800 μL) was added into cyclodextrin solutions (10 mmol/L; 800 mL) and shaken at 25°C for 30 min. Each 15 mL of shaken solution was sealed into a PA bottle and placed at 4, 25 and 37°C, respectively. Samples were taken at 5, 10, 20, 45 and 90 days to extract and determine the off-flavor compounds by SPME-GC–MS.

### 2.5 Predictive modeling

In the data preprocessing phase, the Pandas library is used to read the data and isolate the feature and target variables. Subsequently, the data was normalized to improve the performance of the model. Predictive models were built using traditional statistical predictive models (Polynomial Regression) and deep learning models (Multilayer Perceptron). Sucrose content, citric acid content, pH, temperature, and storage conditions were used as input variables, and peak areas of off-flavor compounds (*α*-terpineol, terpinen-4-ol, and carvone) were used as output layers. Three target variables were used for model training and predictions were made using the trained model. Predictions were normalized back to the original scale by inverse normalization and the predictive accuracy of the models was assessed using R^2^ scores.

### 2.6 Statistical analysis

The experimental data were expressed as the means ± standard deviations (*n* = 3). The significant difference was analyzed via a Multiple-range Duncan’s test (*p* < 0.05) performed with the methods reported previously (22).

## 3 Results and discussion

### 3.1 Effect of *β*-CD on the citrus juice

#### 3.1.1 Sensory evaluation

**Figure 1:**
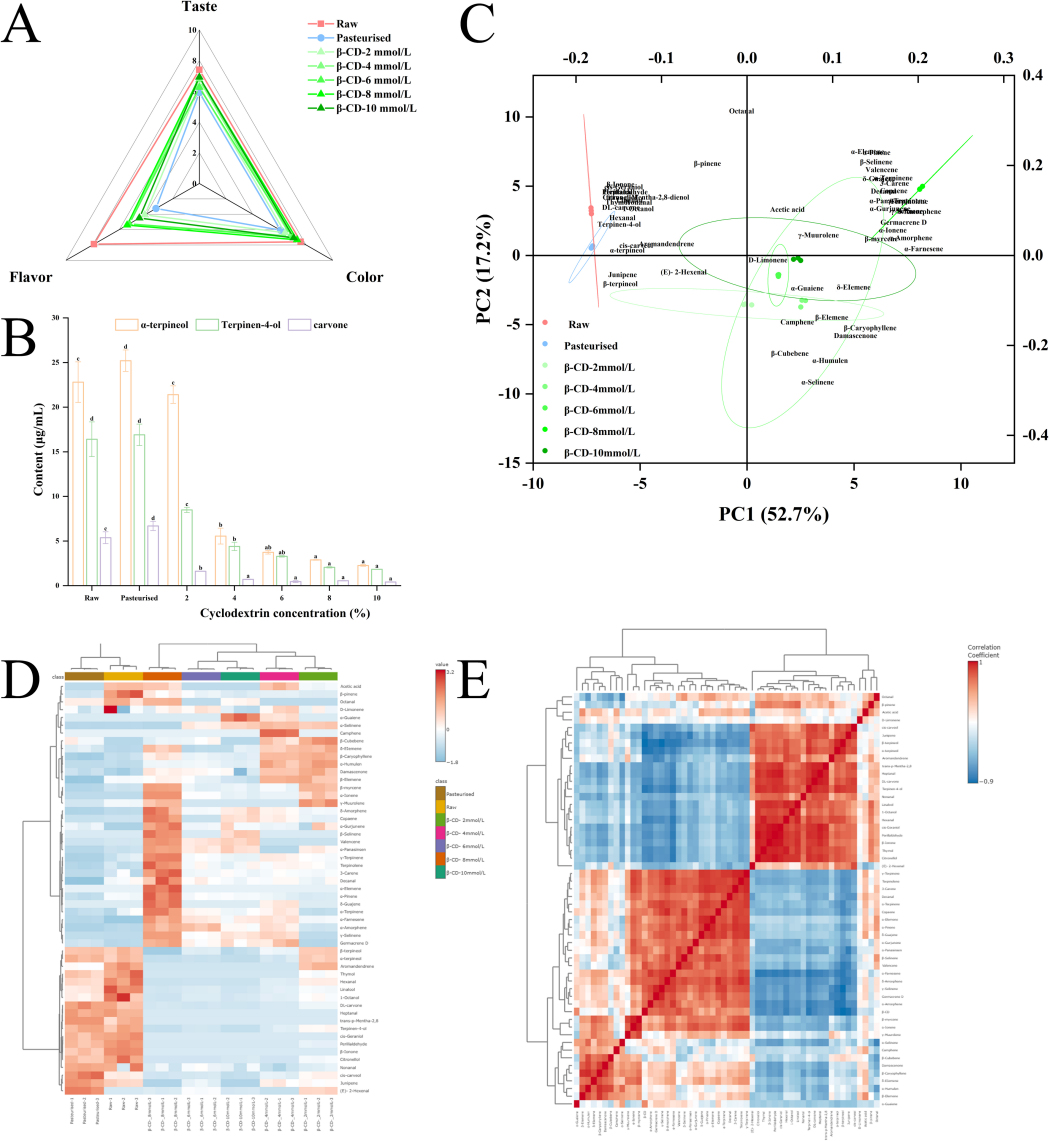
**(A)** Sensory evaluation of citrus juice; **(B)** Changes in the contents of three off-flavor; **(C)** Principal component analysis of volatile substances; **(D)** Heatmap of volatile substances; **(E)** correlation heat map. The different letters in the same line indicate significant differences by Duncan’s multiple range tests (*p* < 0.05).

The sensory characteristics of the citrus juice were presented in Figure 1A. The scores of the indicators decreased significantly after pasteurization of citrus fruit juice. And the indexes were improved after the addition of *β*-CD, indicating that the *β*-CD addition could improve the flavor of citrus juices. The highest scores were obtained with the addition at 4 mmol/L. The result indicated that *β*-CD were effective in encapsulating the off-flavor compounds in citrus juices. It was worth noting that excessive additions of *β*-CD (10 mmol/L) resulted in lower flavor scores, which might be due to the encapsulation effect of *β*-CD on other flavor compounds. In addition, *β*-CD -added citrus juices were rated better than non-*β*-CD -added citrus juices in terms of taste and color, suggesting that *β*-CD not only improved the flavor characteristics of citrus juices, but also the taste and appearance characteristics. This was similar to the results of previous studies (9, 11).

#### 3.1.2 Volatile compounds

Table 1 and heatmap of volatile compounds (Figure 1D) showed the 53 volatile compounds detected in citrus juice. These compounds were alkenes, aldehydes, alcohols, and ketones. Of these, 32 volatile compounds could be defined as key volatile compounds because their OAV was >1 (15). Odor description and threshold were obtained from www.perflavory.com, www.femaflavor.org, www.odour.org.uk and previous literatures (23–26). Overall, citrus juices showed a decrease in flavor after pasteurization and an increase in flavor after *β*-CD treatment. This result was consistent with the results of the sensory evaluation (Figure 1A). Principal component analysis (PCA) can assess the correlation between multiple variables by evaluating the intensity of volatile compounds. The result of significant changes in citrus juices after pasteurization and *β*-CD treatment can also be derived from the PCA of volatile compounds (Figure 1C).

**Table 1:** Content of volatile compounds of citrus juice.

No.	Volatile compounds (μg/mL)	Odor description	Threshold (μg/mL)	R	P	*β*-CD (mmol/L)					R	P	*β*-CD (mmol/L)				
						2	4	6	8	10			2	4	6	8	10
Alkenes																	
1	3-Carene	Lemon, resin	0.77	1.11 ± 0.35^ab^	0.77 ± 0.01^a^	1.49 ± 0.04^bc^	1.56 ± 0.07^c^	1.44 ± 0.03^bc^	3.42 ± 0.29^d^	1.52 ± 0.13^bc^	1.44	1	1.94	2.03	1.87	4.44	1.97
2	Aromandendrene	–	–	2.55 ± 0.05^c^	0.19 ± 0.01^a^	0.46 ± 0.03^b^	–	–	–	–	–	–	–	–	–	–	–
3	Camphene	Camphor	0.15	–	–	–	0.23 ± 0.01^a^	–	–	–	–	–	–	1.52	–	–	–
4	Copaene	Wood, spice	0.006	4.27 ± 0.02^a^	0.13 ± 0.03^a^	1.14 ± 0.11^b^	2.57 ± 0.23^c^	0.15 ± 0.01^a^	6.45 ± 0.56^e^	3.39 ± 0.24d	45	21	190	428.33	25.67	1,075	565
5	D-Limonene	Lemon, orange	0.2	5.8 ± 72.1^a^	439.9 ± 11.3^a^	452.02 ± 9.3^a^	477.2 ± 12.3^a^	468.08 ± 6.77^a^	455.89 ± 13.44^a^	427.5 ± 9.45a	2,359	2199.5	2260.1	2,386	2340.4	2279.45	2137.5
6	Germacrene D	Wood, spice	–	6.2 ± 0.02^a^	0.43 ± 0.03^a^	0.15 ± 0.02^a^	1.72 ± 0.34^c^	1.06 ± 0.11^b^	2.11 ± 0.11^d^	1.26 ± 0.21b	–	–	–	–	–	–	–
7	Junipene	–	–	7.47 ± 0.03^c^	0.68 ± 0.04^d^	0.31 ± 0.02^b^	0.36 ± 0.04^b^	0.21 ± 0.02^a^			–	–	–	–	–	–	–
8	Terpinolene	Pine	0.2	8.38 ± 0.51^a^	1.3 ± 0.09^a^	5.01 ± 0.45^b^	5.33 ± 0.45^b^	4.83 ± 0.56^b^	11.86 ± 1.09^c^	4.56 ± 0.89b	11.9	6.5	25.05	26.65	24.15	59.3	22.8
9	Valencene	Green, oil	–	9.74 ± 0.03^b^	0.75 ± 0.03^ab^	0.27 ± 0.03^a^	-	13.81 ± 1.09^c^	28.18 ± 0.58^e^	17.69 ± 1.14d	–	–	–	–	–	–	–
10	α-Pinene	Pine, turpentine	0.041	10.63 ± 0.09^c^	0.59 ± 0.08^a^	1.09 ± 0.04^b^	1.39 ± 0.15^bc^	1.7 ± 0.12^c^	4.97 ± 0.35^d^	1.69 ± 0.06c	39.76	14.39	26.59	33.9	41.46	121.22	41.22
11	β-pinene	Pine, resin, turpentine	0.14	11.32 ± 0.28^d^	0.36 ± 0.03^a^	0.5 ± 0.03^ab^	0.58 ± 0.04^ab^	0.69 ± 0.08^b^	1.02 ± 0.14^c^	0.48 ± 0.03ab	16.57	2.57	3.57	4.14	4.93	7.31	3.44
12	α-Terpinene	Lemon	0.08	12.96 ± 0.46^ab^	0.53 ± 0.08^a^	0.52 ± 0.04^a^	1.74 ± 0.13^d^	1.56 ± 0.05^cd^	3.34 ± 0.24^e^	1.25 ± 0.11bc	12	6.63	6.44	21.75	19.5	41.75	15.63
13	γ-Terpinene	Gasoline, turpentine	1	13.2 ± 5.1^b^	8.63 ± 0.23^a^	37.11 ± 1.45^d^	46.63 ± 1.78^e^	36.64 ± 1.24^d^	89.36 ± 1.88^f^	31.12 ± 0.87c	24.2	8.63	37.11	46.63	36.64	89.36	31.12
14	α-Elemene	–	–	–	–	–	–	–	0.59 ± 0.04^a^	–	–	–	–	–	–	–	–
15	δ-EIemene	Wood	–	15.51 ± 0.02^a^	0.67 ± 0.04^b^	1.28 ± 0.06^d^	1.15 ± 0.05^c^	0.52 ± 0.01^a^	1.19 ± 0.06^cd^	0.65 ± 0.08b	–	–	–	–	–	–	–
16	β-Elemene	Herb, wax, fresh	–	16.97 ± 0.06^cd^	1.23 ± 0.03^a^	8.41 ± 0.81^e^	9.07 ± 0.13^e^	4.57 ± 0.49^c^	5.76 ± 0.29^d^	3.15 ± 0.25b	–	–	–	–	–	–	–
17	α-Selinene	Amber	0.001	17.21 ± 0.01^a^	0.18 ± 0.02^a^	6.5 ± 0.3^c^	7.2 ± 0.21^d^	4.44 ± 0.32^b^	–	6.1 ± 0.23c	210	180	6,500	7,200	4,440	–	6,100
18	β-Selinene	Herb	–	–	–	–	–	–	6.34 ± 0.43^b^	3.8 ± 0.45a	–	–	–	–	–	–	–
19	γ-Selinene	Wood	–	–	0.13^a^	–	5.68 ± 0.36^d^	3.35 ± 0.24^b^	8.96 ± 0.03^e^	4.86 ± 0.23c	–	–	–	–	–	–	–
20	α-Amorphene	–	–	20.18 ± 0.03^a^	-	0.48 ± 0.03^b^	1.62 ± 0.16^e^	2.25 ± 0.13^d^	2.48 ± 0.18^e^	1.22 ± 0.09c	–	–	–	–	–	–	–
21	δ-Amorphene	Fruity	–	21.46 ± 0.02^a^	0.16 ± 0.02^a^	1.74 ± 0.09^b^	2.41 ± 0.13^bc^	1.83 ± 0.03^b^	5.14 ± 0.81^d^	2.96 ± 0.34c	–	–	–	–	–	–	–
22	α-Farnesene	Wood, sweet	–	–	0.61 ± 0.04^a^	1.78 ± 0.04^b^	2.75 ± 0.13^d^	2.63 ± 0.09^cd^	4.17 ± 0.32^e^	2.31 ± 0.34c	–	–	–	–	–	–	–
23	α-Panasinsen	–	–	23.17 ± 0.02^a^	-	0.72 ± 0.06^b^	-	0.77 ± 0.04^b^	1.39 ± 0.03^d^	0.89 ± 0.09c	–	–	–	–	–	–	–
24	α-Ionene	Fruit, licorice	0.0106	–	–	0.34 ± 0.04^c^	0.15 ± 0.01^a^	0.19^b^	0.53 ± 0.01^d^	0.13 ± 0.02a	–	–	31.6	13.77	18.11	49.62	11.89
25	α-Guaiene	Wood, balsamic	–	–	–	–	0.13 ± 0.01^a^	–	–	0.29 ± 0.02b	–	–	–	–	–	–	–
26	δ-Guajene	–	–	–	–	–	0.3 ± 0.04^a^	–	0.81 ± 0.05^b^	–	–	–	–	–	–	–	–
27	α-Gurjunene	Wood, balsamic	–	–	0.16 ± 0.02^a^	0.21 ± 0.12^a^	0.16 ± 0.01^a^	0.17 ± 0.01^a^	0.53 ± 0.05^b^	0.25 ± 0.08a	–	–	–	–	–	–	–
28	α-Humulen	Wood	120	28.23 ± 0.07^a^	0.2 ± 0.01^a^	3.77 ± 0.23^d^	3.32 ± 0.45^c^	1.25 ± 0.05^b^	1.21 ± 0.03^b^	0.95 ± 0.07b	0	0	0.03	0.03	0.01	0.01	0.01
29	β-myrcene	Balsamic, must, spice	0.0012	29.88 ± 0.37^b^	2.37 ± 0.09^a^	26.06 ± 1.12^e^	11.61 ± 1.12^d^	9.69 ± 0.99^c^	30.05 ± 0.44^f^	10.07 ± 0.89 cd	4,900	1975	21716.67	9,675	8,075	25041.67	8391.67
30	β-Cubebene	Citrus, fruit	–	–	0.21 ± 0.03^a^	0.51 ± 0.04	0.34 ± 0.07^b^	0.21 ± 0.03^a^	0.15 ± 0.02^a^		–	–	–	–	–	–	–
31	β-Caryophyllene	Wood, spice	0.064	31.46 ± 0.06^b^	0.14 ± 0.04^a^	2.04 ± 0.05^e^	1.92 ± 0.01^e^	0.85 ± 0.06^c^	1.34 ± 0.11^d^	0.73 ± 0.04c	7.19	2.19	31.88	29.95	13.22	20.94	11.41
32	γ-Muurolene	Herb, wood, spice	–	32.17^a^	–	0.72 ± 0.05^c^	–	–	0.55 ± 0.04^b^	–	–	–	–	–	–	–	–
Aldehydes																	
33	Hexanal	Grass, tallow, fat	0.005	33.72 ± 0.44^e^	1.97 ± 0.09^d^	1.52 ± 0.03^c^	0.44 ± 0.03^b^	0.06 ± 0.01^a^	–	–	744	394.4	304	88.8	11.6	–	–
34	Heptanal	Fat, citrus, rancid	0.003	34.4 ± 0.03^a^	0.41 ± 0.03^a^	–	–	–	–	–	134	136.67	–	–	–	–	–
35	Octanal	Fat, soap, lemon, green	0.0007	35.3 ± 0.62^b^	2.13 ± 0.02^a^	–	–	–	3.56 ± 0.05^b^	1.94 ± 0.12a	4714.29	3042.86	–	–	–	5085.71	2771.43
36	Nonanal	Fat, citrus, green	0.0011	36.19 ± 0.8^a^	2.58 ± 0.04^a^	1.22 ± 0.04^b^	0.91 ± 0.12^b^	1.46 ± 0.08^b^	1.19 ± 0.14^b^	1.23 ± 0.21b	1990.91	2345.45	1109.09	822.73	1327.27	1081.82	1118.18
37	Decanal	Soap, orange peel, tallow	0.0049	37.19 ± 0.06^b^	0.45 ± 0.03^a^	1.14 ± 0.11^b^	1.25 ± 0.05^b^	1.29 ± 0.08^b^	2.11 ± 0.34^c^	1.14 ± 0.05b	242.86	91.84	232.65	255.1	263.27	430.61	232.65
38	(E)- 2-Hexenal	Green, leaf	0.11	38.42 ± 0.01^a^	4.5 ± 0.19^d^	2.5 ± 0.09^c^	–	1.22 ± 0.03^b^	0.53 ± 0.05^a^	0.39 ± 0.04a	3.82	40.91	22.73	–	11.09	4.82	3.55
39	Perillaldehyde	Fat	0.03	39.48 ± 0.05^c^	1.09 ± 0.1^b^	0.18 ± 0.02^a^	–	–	–	–	49.33	36.33	5.97	–	–	–	–
Alcohols																	
40	1-Octanol	Moss, nut, mushroom	0.1258	40.54 ± 1.9^c^	2.74 ± 0.24^b^	2.01 ± 0.21^ab^	1.03 ± 0.03^a^	0.91 ± 0.06^a^	0.38 ± 0.03^a^	0.61 ± 0.09a	51.99	21.78	15.98	8.19	7.22	3.02	4.81
41	Linalool	Flower, lavender	0.006	41.5 ± 7.1^d^	28.6 ± 1.9^c^	16.43 ± 0.92^b^	9.91 ± 0.78^a^	7.84 ± 0.47^a^	4.79 ± 0.45^a^	4.17 ± 0.65a	10916.67	4766.67	2738.33	1651.67	1306.67	798.33	695
42	cis-Geraniol	Rose, geranium	0.04	42.22 ± 0.01^b^	0.18 ± 0.02^a^	–	–	–	–	–	5.5	4.5	–	–	–	–	–
43	α-terpineol	Oil, anise, mint	0.35	43.8 ± 2.27^c^	25.2 ± 1.2^d^	21.4 ± 0.98^c^	5.55 ± 0.89^b^	3.73 ± 0.19^ab^	2.88 ± 0.04^a^	2.26 ± 0.09a	65.14	72	61.14	15.86	10.66	8.23	6.46
44	β-terpineol	Must	0.35	44.1 ± 0.17^c^	2.1 ± 0.04^c^	2 ± 0.03^c^	0.95 ± 0.04^b^	0.8 ± 0.02^b^	–	0.42 ± 0.08a	6	6	5.71	2.71	2.27	–	1.2
45	Citronellol	Rose	0.062	45.23 ± 0.48^d^	2.29 ± 0.23^c^	0.71 ± 0.06^b^	–	–	–	0.15 ± 0.05a	52.1	36.94	11.42	–	–	–	2.35
46	Terpinen-4-ol	Turpentine, nutmeg, must	0.34	46.4 ± 1.95^d^	16.9 ± 1.2^d^	8.48 ± 0.29^c^	4.39 ± 0.47^b^	3.28 ± 0.08^ab^	2.05 ± 0.07^a^	1.83 ± 0.02a	48.24	49.71	24.94	12.91	9.65	6.03	5.38
47	cis-carveol	Caraway	16.4	47.71 ± 0.09^d^	5.8 ± 0.45^e^	2.48 ± 0.12^d^	1.39 ± 0.13^c^	1.05 ± 0.08^bc^	0.91 ± 0.06^ab^	0.59 ± 0.06a	0.17	0.35	0.15	0.08	0.06	0.06	0.04
48	trans-p-Mentha-2,8-dienol	–	–	48.91 ± 0.37^a^	2.55 ± 0.01^b^	–	–	–	–	–	–	–	–	–	–	–	–
Ketones																	
49	DL-carvone	Mint, basil, fennel	2.4	49.37 ± 0.66^c^	6.68 ± 0.51^d^	1.61 ± 0.01^b^	0.7 ± 0.03^a^	0.45 ± 0.1^a^	0.55 ± 0.03^a^	0.41 ± 0.02a	2.24	2.78	0.67	0.29	0.19	0.23	0.17
50	Damascenone	Apple. rose, honey	–	50.23 ± 0.04^a^	0.34 ± 0.03^a^	1.03 ± 0.08^c^	0.93 ± 0.02^c^	0.64 ± 0.03^b^	0.7 ± 0.05^b^	0.37 ± 0.25a	–	–	–	–	–	–	–
51	β-Ionone	Seaweed, violet, flower, raspberry	0.006	51.34 ± 0.01^b^	0.24 ± 0.02^a^	–	–	–	–	–	57	40	–	–	–	–	–
Others																	
52	Thymol	Thyme	1.7	52.65 ± 0.25^c^	1.05 ± 0.12^b^	0.48 ± 0.05^a^	–	–	–	–	0.97	0.62	0.28	–	–	–	–
53	Acetic acid	Sour	99	53.23 ± 0.01^c^	-	0.1 ± 0.01^a^	0.24 ± 0.02^b^	–	0.19 ± 0.02^b^	–	0	–	0	0	–	0	–

R, Raw; P, Pasteurized; OVA, odor activity values; “-”: no detection or unable to be calculated. Different letters in the same line indicate significant differences by Duncan’s multiple range tests (*p* < 0.05).

Alkenes were the main volatile compounds in citrus juices (27). There were 12 key volatile compounds found in the samples, including 3-Carene, camphene, copaene, D-limonene, terpinolene, *α*-pinene, β-pinene, α-terpinene, *γ*-terpinene, α-selinene, α-ionene, α-humulen, β-myrcene and β-caryophyllene (Table 1). Isopentenyl pyrophosphate (IPP) and dimethylallyl pyrophosphate (DMAPP), produced via the mevalonic acid pathway and the methylerythritol phosphate pathway, were common precursors for the biosynthesis of these alkenes (28, 29). IPP and DMAPP could be interconverted by isopentenyl diphosphate isomerase (IDI). IPP and DMAPP were catalyzed by farnesyl diphosphate synthase to produce farnesyl pyrophosphate or by geranyl diphosphate synthase to produce geranyl diphosphate, which were then synthesized by terpene synthase to produce various terpenes (28, 29). D-Limonene was the most abundant in all samples, accounting for more than 60% of the total content of all volatile components. However, the highest OAV value was *β*-myrcene because of its lower threshold. The alkene content of citrus juices was significantly reduced after pasteurization. It could be that these alkenes escaped during the heating process. With the addition of *β*-CD, the olefin content returned to its original level or even increased slightly.

Aldehydes, alcohols and ketones were also important components of volatile flavor compounds in citrus juice (27, 30). There were 7 aldehydes, 7 alcohols and 2 ketones identified as volatile compounds. These compounds, especially aldehydes such as hexanal, heptanal, octanal, nonanal, decanal and (E)-2-hexenal were produced by lipid oxidation and were off-flavor compounds in citrus juices, where they impart an undesirable sensation of fat, tallow, or grease (8, 31, 32). These aldehydes were produced mainly from the degradation products of lipids such as oleic, linoleic, linolenic, arachidonic, eicosapentaenoic and docosahexaenoic acids through the formation of hydroperoxides by the enzyme lipoxygenase, which were then catalytically produced by the enzyme hydroperoxide lyase (15, 33). Some aldehydes are then reduced to the corresponding alcohols catalyzed by ethanol dehydrogenase (15, 33). Most of these compounds were significantly reduced by the addition of cyclodextrins. This result suggested that *β*-CD had an encapsulating effect on aldehydes, alcohols and ketones, which reduced the off-flavor of citrus juice (10). It was noteworthy that aroma compounds would be encapsulated by *β*-CD along with off-flavor compounds. Excessive addition of cyclodextrins might cause a decrease in sensory evaluation (Figure 1A).

The correlation heat map (Figure 1E) demonstrated a strong negative correlation between *β*-CD and the three off-flavor compounds, suggesting that these compounds might decrease with the increase of *β*-CD due to their encapsulation by *β*-CD. The changes of three off-flavor compounds in citrus juice before and after pasteurization and after addition of *β*-CD were shown in Figure 1B. After pasteurization, the contents of off-flavor compounds were slightly increased but not significantly different, however, the contents decreased significantly after the addition of cyclodextrin. With the increases of *β*-CD concentrations, the contents of all three off-flavor compounds decreased gradually. The decreases were greater 4 mmol/L. At concentrations >4 mmol/L, the contents continued to decrease, but not to a significant extent. The results indicated that the appropriate concentration of the *β*-CD was 4 mmol/L, which had a good encapsulation effect on the three off-flavor compounds.

#### 3.1.3 Physicochemical indexes

The pH was a fundamental and important parameter affecting food. Components such as titratable acids and soluble solids contained in foods can had a significant impact on their taste and flavor (34). The changes in pH, soluble solids and titratable acids of citrus juice were shown in the Table 2. With the addition of *β*-CD, pH (3.5–3.6) and titratable acid (0.7–0.8) changed less, while soluble solids increased significantly (9.5–10.5). This result suggested that *β*-CD had a limited effect on citrus juice pH and acidity, but might significantly increase the sweetness of citrus juice. This might account for the improved taste in the sensory evaluation (Figure 1A).

**Table 2:** Physicochemical indexes and nutrients of citrus juice.

Indexes	Raw	Pasteurized	*β*-CD (mmol/L)				
			2	4	6	8	10
pH	3.54 ± 0.01^c^	3.57 ± 0.00^a^	3.59 ± 0.02^bc^	3.57 ± 0.01^c^	3.57 ± 0.00^bc^	3.56 ± 0.01^bc^	3.53 ± 0.01^ab^
Titratable acid	0.768 ± 0.01^ab^	0.789 ± 0.02^ab^	0.725 ± 0.03^b^	0.789 ± 0.02^a^	0.789 ± 0.02^b^	0.789 ± 0.03^a^	0.832 ± 0.01^b^
Soluble solids (°Brix)	9.5 ± 0.1^ab^	9.5 ± 0.2^a^	10.0 ± 0.2^a^	10.0 ± 0.1^b^	9.5 ± 0.2^b^	10.5 ± 0.1^b^	9.5 ± 0.1^c^
Vitamin C (mg/100 g)	25.1 ± 0.22^f^	24.2 ± 0.32^e^	24.6 ± 0.11^cd^	23.3 ± 0.32^de^	24.2 ± 0.32^b^	22.6 ± 0.38^bc^	24.87 ± 0.22^a^
Total flavonoids (g/kg)	3.25 ± 0.01^d^	3.37 ± 0.09^c^	2.98 ± 0.1^cd^	2.72 ± 0.01^b^	3.37 ± 0.09^a^	2.95 ± 0.05^a^	2.8 ± 0.09^b^

Different letters in the same line indicate significant differences by Duncan’s multiple range tests (*p* < 0.05).

#### 3.1.4 Nutrients

Vitamin C and flavonoids were important active ingredients in citrus juice, but they were unstable in nature and could be affected by various factors such as temperature, pressure, light as well as acid (1). Therefore, it was necessary to investigate the effect of *β*-CD addition on Vitamin C and flavonoids (Table 2). Both Vitamin C and flavonoids showed an overall decreasing trend with the addition of *β*-CD. The results showed that vitamin C and flavonoids were encapsulated in *β*-CD along with off-flavor compounds (35, 36). Therefore, attention should be paid to control the amount of *β*-CD added, and the addition ratio should not be too high.

### 3.2 Interactions between *β*-CD and off-flavor compounds

#### 3.2.1 Retention rate, embedding rate, and formation constant

**Figure 2:**
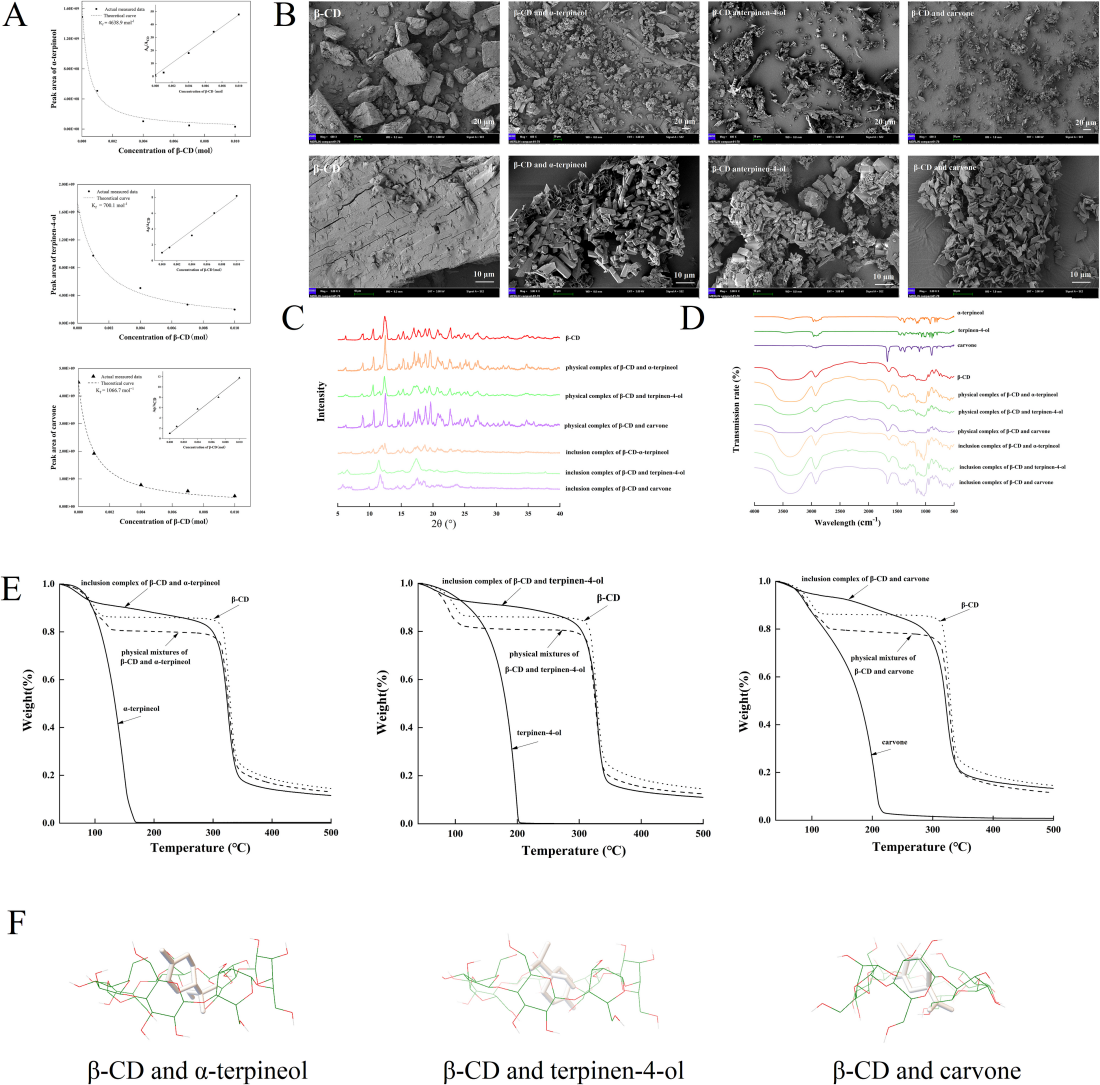
**(A)** Representation of the experimental points obtained for *α*-terpineol, terpinen-4-ol, carvone and *β*-CD compared with theoretical titration curve for a 1:1 complex; **(B)** SEM of *β*-CD and three inclusion complexes; **(C)** FT-IR of guest molecules, *β*-CD, physical mixtures and inclusion complexes; **(D)** XRD of *β*-CD, physical mixtures and inclusion complexes; **(E)** TGA of guests, *β*-CD, physical mixtures and inclusion complexes; **(F)** Possible conformations of *β*-CD-embedded α-terpineol, terpinen-4-ol and carvone.

The retention rate and embedding rate of cyclodextrin to guest molecules were presented in Table 3. The tendency to form inclusion complexes between *β*-CD and these three guest molecules could be inferred from the high retention and embedding rates of *β*-CD for the three guest molecules (17, 19). Among them, *α*-terpineol had the highest retention rate, while carvone had the highest embedding rate. Formation constants could directly reflect the ability and stability of various subjects to encapsulate objects (21). As shown by the results of the formation constant measurements (Figure 2A), *β*-CD was able to form 1:1 inclusion complexes with three off-flavor compounds. In comparison with the previous literature, this result suggests that the inclusion complexes formed by CD and guest molecules had high degree of stability (19, 21).

**Table 3:** Retention rate and embedding rate of cyclodextrin to guest molecules.

Off-flavor compounds	Retention rate of (%)	Embedding rate (%)
α-terpineol	97.9 ± 0.22	40.62 ± 0.92
terpinen-4-ol	89.1 ± 0.69	42.14 ± 2.06
carvone	82.94 ± 0.26	75.05 ± 9.16

#### 3.2.2 SEM analysis

The scanning electron microscopy results of *β*-CD and the three inclusion compounds magnified 600 and 3,000 times, respectively, reveal that the size as well as the surface morphology of the simple *β*-CD as well as the inclusion compounds of *β*-CD with the three guest molecules were very different (Figure 2B). All four compounds show a distinct crystalline state, but the volume of the three inclusion complexes was significantly smaller than that of *β*-CD. The surface of *β*-CD was loose, irregular and uneven, suitable for encapsulating small molecules. The surfaces of the three inclusion complexes were smoother and flatter and had a somewhat higher degree of crystal aggregation relative to *β*-CD. Among them, the inclusion complexes of *β*-CD and *α*-pinitol as well as *β*-CD and 4-terpineol showed a regular cubic morphology, while the inclusion complexes of *β*-CD and carvone showed a regular lamellar structure with a compact and smooth surface. The *β*-CD and the inclusion complexes were not the same in terms of apparent morphology, but it could be observed that their apparent morphology before and after cyclodextrin inclusion had become smooth and tight, and their shapes were a little bit more regular, which could indicate that the inclusion complexes were a new phase of the compound, and the state of existence was more stable (21).

#### 3.2.3 XRD analysis

Many sharp diffraction peaks appeared in all seven samples, indicating the presence of many different mimetic states (Figure 2C). There was no significant difference in the number and position of diffraction peaks for the three physical mixtures and *β*-CD, except for a slight difference in intensity. These results suggested that the guest molecules could not enter the cavity of *β*-CD by physical mixing, and therefore the crystalline shape of cyclodextrins was not changed. In contrast, the diffraction peaks of *β*-CD and the three inclusion compounds were significantly different, suggesting the creation of a new phase. Among them, *β*-CD showed diffraction peaks at 2θ = 4.50, 8.88, 10.6, 12.46, 16.96, 20.72, 22.7, 24.28, and 27.05°, while the three inclusion compounds did not have these diffraction peaks. Among them, the inclusion complex of *β*-CD and *α*-terpineol had the highest number of diffraction peaks ranging from 2θ = 7.09 to 22.77°, but the intensities of these peaks were weak. The diffraction peaks of the inclusion complex of *β*-CD and terpinen-4-ol were concentrated at 2θ = 5.59–20.7°, while the inclusion complex of *β*-CD and carvone showed new diffraction peaks at 2θ = 6.52, 9.38, 14.88, 18.1, 25.7, 30.1°, etc. In addition, the number of diffraction peaks of the inclusion complexes was significantly less than that of *β*-CD, but the peaks were broader, suggesting the crystallinity of inclusion complexes was lower than that of *β*-CD.

#### 3.2.4 FT-IR analysis

Since the absorption peaks of cyclodextrins were shifted or the intensity was altered by the presence of guest molecules, FT-IR was often utilized to demonstrate the formation of inclusion complexes (Figure 2D). The absorption peaks of O-H (3375.1 cm^−1^), C-H (2929.18 cm^−1^), H-O-H (1,643 cm^−1^), and C-C (1156.91 and 1028.46 cm^−1^) were mainly present in *β*-CD; the absorption peaks of O-H (3,400 cm^−1^), C-H (2,970 and 2,913 cm^−1^), and C=C (1,374 cm^−1^) were mainly present in *α*-terpineol; terpinen-4-ol was mainly present in -OH (3,430 cm^−1^), -CH2 (2,960 and 2,820 cm^−1^), and -C-OH (1,430 cm^−1^) absorption peaks; whereas carvone was mainly present in C-H (2922.28 cm^−1^), C=O (1669.90 cm^−1^), C=C (1365.88 and 1245.57 cm^−1^), and C-C (1109.81 cm^−1^) absorption peaks. Similar to the results of XRD, there was no significant difference in the number and location of diffraction peaks for the three physical mixtures and *β*-CD, except for a slight difference in intensity. In contrast, the absorption peaks of the inclusion complexes were significantly different. The O-H characteristic peak of α-terpineol was masked by the broader O-H characteristic peak of *β*-CD in the inclusion complex. The absorption peak of terpinen-4-ol (1,460 cm^−1^) was found in the inclusion complex of *β*-CD and 4-terpinol, and the characteristic peak of terpinen-4-ol at 1430 cm-1 was shifted to 1,413 cm^−1^. Similarly, the absorption peak of carvone (1669.90 cm^−1^) was also shifted. The FT-IR results indicated the formation of a new phase, proving that *β*-CD interacted with the guest molecules to form inclusion complexes (19, 37). In addition, the absorption peaks of *β*-CD at 1670, 1030 and 890 cm^−1^ have the same intensity shape and position as those of the inclusion at this location, i.e., new absorption peaks did not appear. This result indicated that no new chemical bond was formed during the process of *β*-CD inclusion and the structure of *β*-CD was not changed. The forces between the guest compounds and cyclodextrins were non-covalent bonds, mainly including hydrogen bonds, hydrophobic interactions and van der Waals forces.

#### 3.2.5 TGA analysis

Thermogravimetric analysis was used to evaluate the thermal stability and volatility of guest molecules in inclusion compounds (Figure 2E). Due to the extreme volatility of guest molecules, *α*-terpineol, terpinen-4-ol and carvone had a rapid mass loss of 99% at 40–170°C, 40–170°C and 40–220°C, respectively. There were two stages of weight loss in *β*-CD. Stage I (50–100°C) and Stage II (310°C) were due to water evaporation (13.59%) and decomposition of *β*-CD, respectively. There were two stages of weight loss exist for the physical mixtures. Stage I (40–130°C) was mainly due to volatilization of the guest molecules and water evaporation of *β*-CD, while Stage II (300°C) was due to decomposition of *β*-CD. There was a high degree of similarity between the decomposition curves of physical mixtures and *β*-CD, indicating that the guest molecules and *β*-CD were simply physically mixed and no inclusion complex was formed. The weight loss of the inclusion was divided into three stages with decomposition curves very different from those of the corresponding physical mixtures. In the stage I of evaporation of water and volatilization of the guest molecules, the weight loss of the inclusion complexes (13.70, 13.92, and 14.43% for *α*-terpineol, terpinen-4-ol and carvone, respectively) was significantly less than that of the physical mixtures (19.4, 20.19, and 21.39% for *α*-terpineol, terpinen-4-ol and carvone, respectively). The weight loss in stage II was due to the continued volatilization of the guest molecules in *β*-CD at a rate significantly lower than in the physical mixtures. The weight loss of the inclusion complexes in the stage III was also mainly due to the decomposition of *β*-CD, but the maximum decomposition temperatures of the three inclusion complexes (280, 328 and 325°C for α-terpineol, terpinen-4-ol and carvone, respectively) were significantly different from that of *β*-CD (312°C). This might be due to the entry of guest molecules into the *β*-CD cavity, which affected the thermal stability of *β*-CD. These changes indicated that the guest molecules all interact with *β*-CD to form inclusion complexes (20).

#### 3.2.6 Molecular docking analysis

The possible structures of the inclusion complexes of CD and guest molecules were visualized in three dimensions using molecular docking, further validating the results of the above experiments. The theoretical models of the inclusion complexes formed by *β*-CD and three guest molecules were shown in Figure 2F. The computationally calculated binding energy of the *β*-CD and α-terpineol, terpinen-4-ol and carvone inclusion complex were −3.49, −3.24, and −3.55 kcal·mol^−1^, respectively. Multiple conformations of the guest molecule entering *β*-CD might have been observed due to their conformational similarity and similar binding energies. These models demonstrate that hydrophobic forces play a major contribution in inclusion formation (21). In addition, *β*-CD could be linked to terpinen-4-ol through the formation of hydrogen bonds. These results further suggested that CD could form 1:1 inclusion complex with guest molecules.

### 3.3 Encapsulation effect of *β*-CD on the off-flavor compounds under different conditions

#### 3.3.1 Sucrose

**Figure 3:**
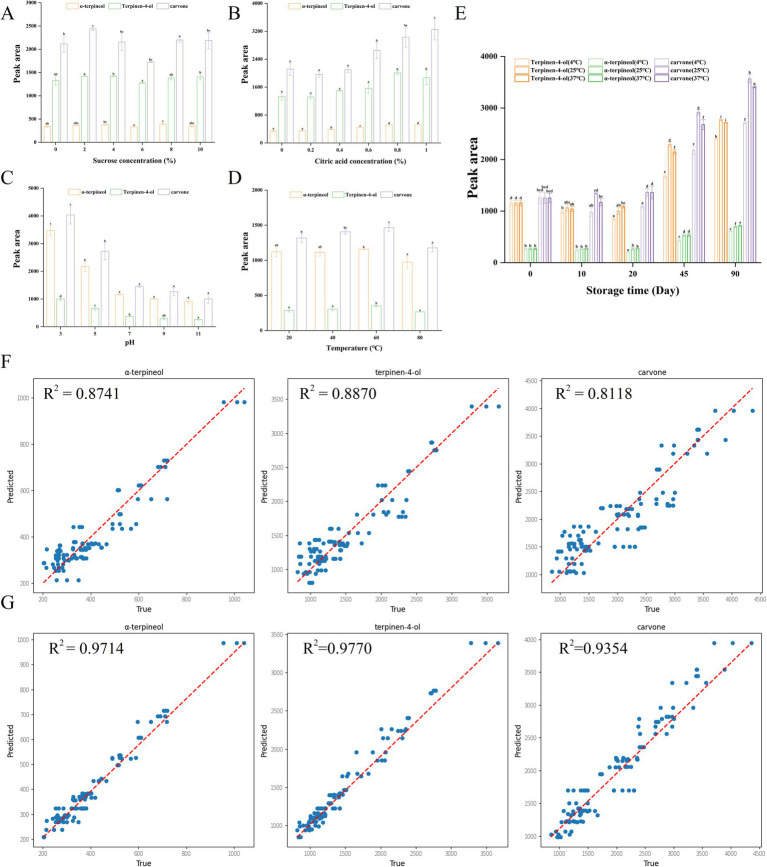
The influence of **(A)** sucrose, **(B)** citric acid, **(C)** pH, **(D)** temperature, and **(E)** storability on the embedding effect of cyclodextrin embedding system; **(F)** polynomial Regression models; **(G)** multilayer perceptron models. Different letters indicate significant differences. The significant differences were obtained from Duncan’s multiple range tests (*p* < 0.05).

Sugars such as sucrose were one of the main components of citrus juice and contributed significantly to the sweetness, content, microbiological stability and color of the food (38). The effect of the addition of sucrose on the embedding effect was shown in Figure 3A. With the increase of sucrose concentration, the peak areas of the three off-flavor compounds in the *β*-CD system showed a trend of decreasing and then increasing, with the minimum peak area obtained at 6%. Overall, however, sucrose had a limited effect on the encapsulation of off-flavor compounds by *β*-CD.

#### 3.3.2 Citric acid

Citric acid was also an important constituent of food and contributed significantly to pH regulation (39). The peak areas of the three off-flavor compounds detected in *β*-CD system increased with increasing citric acid content (Figure 3B). These results suggested that the addition of citric acid was not conducive to the encapsulation of off-flavor compounds by *β*-CD. This may be due to the fact that cyclodextrins were more likely to form less stable complexes with more water-soluble molecules (e.g., sugars and organic acids) than hydrophobic compounds (9, 40).

#### 3.3.3 pH

As shown in Figure 3C, the peak areas of *α*-terpineol, 4-terpinol, and carvone under neutral or alkaline conditions were significantly lower than those under acidic conditions, indicating that neutral or alkaline conditions were more suitable for the embedding of cyclodextrins with off-flavor compounds. This might be due to the fact that cyclodextrins are more inclined to form unstable complexes with the acids in the buffer (9, 40).

#### 3.3.4 Temperature

With the increase of *β*-CD embedding temperature, the peak areas of the three off-flavor compounds showed a trend of increasing and then decreasing (Figure 3D). The minimum peak area was obtained at 80°C, indicating that higher temperatures might contribute to the encapsulation of off-flavor compounds by *β*-CD. The increase in peak area at 20–60°C might be due to the low solubility of *β*-CD, which could not encapsulate the off-flavor compounds well, resulting in volatilization of the off-flavor compounds during the pre-warming process. When the temperature was increased to 80°C, thermal vibrations can help off-flavor compounds to enter the cavity of cyclodextrins (37, 41).

#### 3.3.5 Storability

The release of the three off-flavor compounds from *β*-CD solutions at different storage temperature conditions (4, 25 and 37°C) was shown in Figure 3E. In the *β*-CD embedding system, the release of the three off-flavor compounds at 25 and 37°C storage temperatures was increasing with the increasing of storage time. The peak areas were stable within 20 days and increased significantly after more than 20 days. However, at a storage temperature of 4°C, the peak areas of the three off-flavor compounds decreased and then increased. Among them, *α*-terpineol and Terpinen-4-ol were measured the minimum value on day 20, while carvone were measured the minimum value on day 10. Overall, at normal storage temperatures (4, 25 and 37°C), the cyclodextrin encapsulation systems were able to stably encapsulate the three off-flavor compounds over a 90-day storage period.

### 3.4 Prediction model

There were significant effects of sucrose, citric acid, pH, temperature, and storage conditions on the embedding effect of *β*-CD, but the associated measurements were time-consuming and laborious. Because of its special data processing capabilities, deep learning became an important tool that could predict target data from other intrinsically closely linked data (14). Polynomial Regression was an extension of Linear Regression that fits nonlinear relationships by adding higher order terms. Although essentially a linear model, linear fitting based on polynomials in the input features captures complex nonlinear relationships. In Polynomial Regression, it was critical to choose the appropriate order n, too low for underfitting and too high for overfitting. R^2^ was an important statistic in predictive modeling and was mainly used to measure the goodness of fit or predictive ability of a regression model. The R^2^ of the three off-flavor compounds was <0.8 when *n* = 2. The R^2^ of *α*-pinacol, terpinen-4-ol and carvone, were 0.8741, 0.8870 and 0.8118, respectively, when n was 3 (Figure 3F). After several adjustments, n continued to increase, but there was no significant increase in the R2 of the compounds. The results showed that there was still a large gap between the predicted and true values of the Polynomial Regression model. Multilayer Perceptron was a feed-forward artificial neural network model consisting of an input layer, at least one hidden layer, and an output layer, trained by a back-propagation algorithm, capable of dealing with complex nonlinear relationships. The model architecture employed ReLU activation functions by default to mitigate gradient vanishing in deep layers, with randomized train-test partitioning (80:20 ratio) implemented via train_test_split under a fixed random seed (42), ensuring reproducibility and consistency across experimental trials. The R^2^ of *α*-pinacol, terpinen-4-ol and carvone and carvone 0.9714, 0.9770, and 0.9354, respectively (Figure 3G). The results indicated that the model had good predictive ability and the difference between the predicted and true values was small and within acceptable limits. Multilayer Perceptron models in machine learning had more advantages and potential to handle complex data and predictions than traditional Polynomial Regression models.

## 4 Conclusion

The sensory evaluation of citrus juices was significantly reduced after pasteurization but improved subsequently with the addition of *β*-CD. The GCMS results showed a decrease in the content of aroma compounds and an increase in the content of off-flavor compounds. The addition of *β*-CD improved the sensory evaluation with a significant reduction in off-flavor compounds such as α-terpineol, terpinen-4-ol and carvone. Strong negative correlation between *β*-CD and off-flavor compounds. In addition, there was also a decrease in off-flavor compounds such as hexanal, heptanal, octanal, nonanal, decanal and (E)-2-hexenal. The sensory evaluation also demonstrated *β*-CD not only improved the flavor profile of the citrus juice, but also the taste and appearance characteristics. The results of formation constants suggested that the inclusion complexes had high stability. SEM images showed that the degree of crystal aggregation of the inclusion complexes was higher than that of *β*-CD. Changes in diffraction and absorption peaks in XRD and FT-IR demonstrated the generation of new phases in the inclusion complexes. TGA supported that the inclusion complexes had high stability. Molecular docking demonstrated the possible conformations of *β*-CD and guest molecules forming 1:1 inclusion complex by hydrophobic interaction, van der Waals forces and hydrogen bonding. Sucrose had a limited effect on the embedding of *β*-CD, while citric acid significantly reduces the embedding effect. Neutral, alkaline, and high temperatures conditions were more favorable for embedding. The encapsulation system of *β*-CD was able to stably encapsulate the three off-flavor compounds over a 90-day storage period, with 4°C being more suitable than 25 and 37°C. The Multilayer Perceptron model constructed on the basis of basic indicators had high accuracy and application potential in the prediction of cyclodextrin encapsulation effect.

## Data Availability

The original contributions presented in the study are included in the article/supplementary material, further inquiries can be directed to the corresponding authors.

## Footnotes

^1^ http://www.chemspider.com
